# Output factor comparison of Monte Carlo and measurement for Varian TrueBeam 6 MV and 10 MV flattening filter‐free stereotactic radiosurgery system

**DOI:** 10.1120/jacmp.v17i3.5956

**Published:** 2016-05-08

**Authors:** Jason Y. Cheng, Holly Ning, Barbara C. Arora, Ying Zhuge, Robert W. Miller

**Affiliations:** ^1^ Radiation Oncology Branch, National Cancer Institute, National Institutes of Health Bethesda MD USA

**Keywords:** Monte Carlo, output factors, stereotactic radiosurgery, small field dosimetry

## Abstract

The dose measurements of the small field sizes, such as conical collimators used in stereotactic radiosurgery (SRS), are a significant challenge due to many factors including source occlusion, detector size limitation, and lack of lateral electronic equilibrium. One useful tool in dealing with the small field effect is Monte Carlo (MC) simulation. In this study, we report a comparison of Monte Carlo simulations and measurements of output factors for the Varian SRS system with conical collimators for energies of 6 MV flattening filter‐free (6 MV) and 10 MV flattening filter‐free (10 MV) on the TrueBeam accelerator. Monte Carlo simulations of Varian's SRS system for 6 MV and 10 MV photon energies with cones sizes of 17.5 mm, 15.0 mm, 12.5 mm, 10.0 mm, 7.5 mm, 5.0 mm, and 4.0 mm were performed using EGSnrc (release V4 2.4.0) codes. Varian's version‐2 phase‐space files for 6 MV and 10 MV of TrueBeam accelerator were utilized in the Monte Carlo simulations. Two small diode detectors Edge (Sun Nuclear) and Small Field Detector (SFD) (IBA Dosimetry) were applied to measure the output factors. Significant errors may result if detector correction factors are not applied to small field dosimetric measurements. Although it lacked the machine‐specific $k_{Q_{\text{clin}},Q_{\text{msr}}}^{f_{\text{clin}},f_{\text{msr}}}$ correction factors for diode detectors in this study, correction factors were applied utilizing published studies conducted under similar conditions. For cone diameters greater than or equal to 12.5 mm, the differences between output factors for the Edge detector, SFD detector, and MC simulations are within 3.0% for both energies. For cone diameters below 12.5 mm, output factors differences exhibit greater variations.

PACS number(s): 87.55.k, 87.55.Qr

## I. INTRODUCTION

Although several studies have reported commissioning data for Varian TrueBeam accelerator (Varian Medical Systems, Inc., Palo Alto, CA),^1^, ^2^, ^3^, ^4^ to the best of the authors' knowledge, there are no reported data for Varian's stereotactic radiosurgery system (SRS) with conical collimators. This system utilizes seven conical collimators with diameters of 17.5 mm, 15.0 mm, 12.5 mm, 10.0 mm, 7.5 mm, 5.0 mm, and 4.0 mm. “Small field” size is typically considered as <3 cm×3 cm. The dose measurements of these small field sizes are a significant challenge, due to many factors including source occlusion, detectors size limitation, and lack of lateral electronic equilibrium.^(5)^ For convenience, the various limitations of small field size dosimetry will be termed the “small field effect” in this study. One useful tool in dealing with small field effect is Monte Carlo (MC) simulation. Many recent studies have applied Monte Carlo simulations to examine the output factors of small field sizes used for radiosurgery such as CyberKnife,^6^, ^7^ Gamma Knife,^8^, ^9^ and Brainlab.^(10)^ In addition, numerous studies have used Monte Carlo simulation to examine the accuracy of diode detectors measurements for small field dosimetry.^11^, ^12^, ^13^, ^14^

Monte Carlo simulations require detailed, accurate information on the various components within the accelerator. For proprietary reasons, those components above the Y and X direction collimators (Y jaws, X jaws) were not provided by Varian for TrueBeam. Instead, Varian provided phase‐space files compatible with the International Atomic Energy Agency (IAEA) format just above the Y jaw at 26.7 cm from the source. Although this approach may provide more uniform results across the research community, the limitation of a defined number of original particles may result in simulation bias if not validated. Previous studies have validated the accuracy of version‐1 of the Varian phase‐space files with measurement data.^15^, ^16^ The version‐1 phase‐space files are stored in a cylindrical space. For version‐2, the phase‐space files are stored in a $6.5\times 6.5\;{\text{cm}}^{2}$ two‐dimensional plane located 26.7 cm from the source. Recent studies by Belosi et al.^(17)^ and Rodrigues et al.^(18)^ validated these version‐2 phase‐space files with measurement data for photon and electron beams, respectively. In this study, we report a comparison of the Monte Carlo simulations with measurements by small field detectors of output factors for Varian SRS system with conical collimators for energy of 6 MV flattening filter‐free (6 MV) and 10 MV flattening filter‐free (10 MV) on the Varian TrueBeam accelerator.

## II. MATERIALS AND METHODS

### A. Experimental measurements

In order to validate the Monte Carlo simulation, percentage depth doses (PDD) were measured for 6 MV and 10 MV flattening filter‐free (FFF) beams for a $10\times 10\;{\text{cm}}^{2}$ field at 100 cm SSD. In addition to depth doses, off‐axis ratios (OAR) were measured at depths of maximum dose ($D_{\text{max}}=1.4\;\text{cm}$ for 6 MV; $D_{\text{max}}=2.4\;\text{cm}$ for 10 MV), 10 cm, and 30 cm. These measurements were performed in a three‐dimensional water scanning system, (Blue Phantom^2^, IBA Dosimetry, Memphis, TN) with a PFD‐3G photon diode (IBA Dosimetry) with a sensitive volume of 0.19 mm^3^ and a sensitive diameter of 2 mm perpendicular to the axis of the radiation beam.

Varian's SRS system provides seven conical collimator sizes ranging from 17.5 mm to 4.0 mm. The cone output measurements were performed in the water phantom. To match the commissioning requirements of the SRS system with Varian's Eclipse treatment planning system, the measurements of the seven cones were performed at an SSD of 95 cm with a depth of 5 cm. The output factors were normalized to a reference field size of $5\times 5\;{\text{cm}}^{2}$.

Appropriate selection of detectors based on the size of the sensitive area is very important for obtaining valid measurements of small field sizes. The dimensional resolution of measurement is limited by the two‐dimensional sensitive size of the detectors perpendicular to the axis of the radiation beam. According to a study by Cranmer‐Sargison et al.,^(12)^ there is no significant volume averaging effect for a $5\times 5\;{\text{mm}}^{2}$ field size measured with detector of an active area of less than 1 mm^2^. For this study, two small diode detectors and a cylindrical ionization chamber were used: an Edge detector (Sun Nuclear, Melbourne, FL) with sensitive volume of 0.019 mm^3^ and a sensitive area of $0.8\times 0.8\;{\text{mm}}^{2}$, a Small Field Detector (SFD, IBA Dosimetry) with sensitive volume of 0.017 mm^3^ and a sensitive diameter of 0.6 mm, and an Exradin A16 cylindrical micro‐ionization chamber (Standard Imaging, Middleton, WI) with sensitive volume of 7 mm^3^ and a sensitive length of 3.4 mm which was used as a reference comparison for large cones. A measurement uncertainty of 0.5% (1 SD) is applicable to all experimental measurements in this study.

### B. Monte Carlo simulation

Monte Carlo simulations were performed using the extensively benchmarked EGSnrc (release V4 2.4.0) codes. BEAMnrc was used to simulate the various components within the accelerator and DOSXYZnrc was used to simulate the dose deposited in a water phantom.

#### B.1 10 by 10 cm^2^ and 5 by 5 cm^2^ field size simulation

In this study, version‐2 of the phase‐space files for 6 MV and 10 MV were utilized for Monte Carlo simulation. BEAMnrc was employed to simulate the Y and X collimators of Varian TrueBeam. A total of $5.7\times 10^{10}$ histories were simulated with BEAMnrc transporting the particles from above the Y collimator (the location of version 2 phase‐space) to the bottom of the X collimator for both 6 MV and 10 MV. A phase‐space file was stored just at the bottom of the X collimator at a distance of 44.41 cm from the source for additional simulation involving the SRS cones with BEAMnrc or for direct dose deposition in the water phantom with DOSXYZnrc. For the phase‐space file to be used with SRS cones simulation, BEAMnrc was simulated for a $5\times 5\;{\text{cm}}^{2}$ field size. An additional set of phase‐space files were simulated for $10\times 10\;{\text{cm}}^{2}$ field size for validation of PDD and OAR measurements. The following parameters were applied for all Monte Carlo simulations:

- the global (total) cutoff energy for electron transport (ECUT) and the low‐energy thresholds for the production of secondary bremsstrahlung knock‐on electrons (AE) were set at 0.521 MeV,
- the global (total) cutoff energy for photon transport (PCUT) and the low‐energy threshold for the production of secondary bremsstrahlung photon (AP) were set at 0.01 MeV, and
- no variance reduction techniques, such as Range Rejection, Photon Forcing, Bremsstrahlung Photon Splitting, and Russian Roulette, were applied.

For validation of Monte Carlo methodology, DOSXYZnrc was simulated at 100 cm SSD with field size of $10\times 10\;{\text{cm}}^{2}$ for 6 MV and 10 MV. A water phantom size of $30\times 30\times 40\;{\text{cm}}^{3}$, with voxel size of $2\times 2\times 2\;{\text{mm}}^{3}$ was simulated. The simulations were performed until the relative statistical uncertainties of dose in individual voxels at depth of 10 cm are less than 0.8% at one standard deviation (SD). EGSnrc utilizes the history‐by‐history method to determine the statistical uncertainties.^(19)^ The relative statistical uncertainties of dose along the central axis at depth 30 cm was less than 1.3%. Particles in the phase space files generated by BEAMnrc were sampled only twice. The resampling of particles does not introduce systematic bias because the statistical analysis encoded in EGSnrc is based on the number of independent simulated histories. Walters et al.^(19)^ reported that resampling (“recycling” in EGSnrc) of particles used in BEAMnrc by three times had no effect on the central axis dose uncertainties and resampling 27 times only marginally increased, provided the number of independent simulated histories are the same (no “restarting” in EGSnrc). Depth‐dose ratios at the central axis and off‐axis ratios at depths of maximum dose, 10 cm, and 30 cm were compared with measured data for beam energies of 6 MV and 10 MV.

#### B.2 SRS cone simulation

Simulations of SRS cones were performed for 6 MV and 10 MV. BEAMnrc simulated the particles transport from the stored phase space file exiting the X collimator (at 44.41 cm from the source) to the bottom of the seven conical collimators. The dimensions of the various cone sizes were simulated according to engineering drawings provided by Varian Medical Systems. A total of $5.3\times 10^{9}$ histories were simulated for each cone, and each phase‐space file was stored at a distance equal to the bottom of the cone. DOSXYZnrc was applied to simulate 6 MV and 10 MV photon beams using $5\times 5\;{\text{cm}}^{2}$ as a reference field size for output factors at an SSD of 95 cm. A water phantom of $20\times 20\times 20\;{\text{cm}}^{3}$ and voxel size of $1\times 1\times 1\;{\text{mm}}^{3}$ were simulated. The simulations were performed until the relative statistical uncertainties of dose in the central axis voxel at depth of 5 cm are less than 1% at one standard deviation. Particles in phase space files generated by BEAMnrc were sampled only twice.

For the stereotactic applicators, DOSXYZnrc was applied to simulate the dose deposition in water phantom of the seven SRS cones for 6 MV and 10 MV at 95 cm. A water phantom of $20\times 20\times 20\;{\text{cm}}^{3}$ with voxel size of $0.5\times 0.5\times 0.5\;{\text{mm}}^{3}$ was simulated. The simulations were performed until the relative statistical uncertainties of dose in the central axis voxel at depth of 5 cm are less than 1% at one standard deviation. Particles in phase space files generated by BEAMnrc were sampled no more than five times for all cones. In order to verify that voxel size did not contribute to the partial volume effect, another set of simulations with a voxel size of $0.25\times 0.25\times 0.25\;{\text{mm}}^{3}$ were conducted for cones diameters of 5.0 mm and 4.0 mm. The cost of decreasing the voxel size was an increase in the relative statistical uncertainties, unless additional independent histories were generated. For the same number of histories simulated, the relative statistical uncertainty of the central axis dose at a depth of 5 cm increased to 1.2%.

## III. RESULTS

### A. 10 by 10 cm^2^ field size at 100 SSD

**Figure 1:**
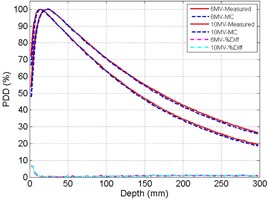
Percentage depth‐dose comparison for 6 MV and 10 MV at 100 SSD with $10\times 10\;{\text{cm}}^{2}$. Solid line represents diode measurement and dashed line represents Monte Carlo simulated result. Percentage difference (delta) of PDD between diode measurement and MC simulation are illustrated by the dash‐dot lines.

Measurement and Monte Carlo simulation data were compared at 100 cm SSD for a field size of $10\times 10\;{\text{cm}}^{2}$ for 6 MV and 10 MV. Figure 1 illustrates the PDD measurement, MC simulation, and percentage differences between the two for 6 MV and 10 MV. Beyond the dose buildup region (>1 cm), the measurement and MC simulation data agree closely with percentage difference of less than 1.5% for both 6 MV and 10 MV. The increased differences in the dose buildup regions (<1 cm) for both energies may be due to limitations in performing accurate measurements, as noted by the Report of AAPM Task Group No. 105.^(20)^ The off‐axis ratios for 6 MV and 10 MV beams using a field size of $10\times 10\;{\text{cm}}^{2}$ at 100 cm SSD and depths of $D_{\text{max}}$, 10 cm, and 30 cm, are shown in 2, 3, respectively. Figure 4 illustrates the percentage differences in OAR between measurements and simulations at 6 MV and 10 MV. Outside of the high gradient profile regions (field edge), the percentage differences of OAR between measurement and simulation are in good agreement (less than 1.5% deviation) for both 6 MV and 10 MV. Within the high gradient profile regions, the percentage differences are less than 3% for both energies.

**Figure 2:**
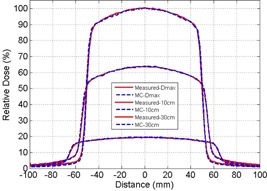
Off‐axis ratio comparison for 6 MV at depths of maximum dose, 10 cm, and 30 cm for 100 SSD with $10\times 10\;{\text{cm}}^{2}$. Solid line represents diode measurement and dashed line represents Monte Carlo simulated result.

**Figure 3:**
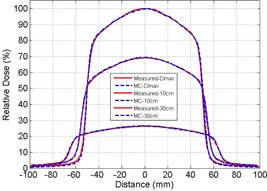
Off‐axis ratio comparison for 10 MV at depths of maximum dose, 10 cm, and 30 cm for 100 SSD with $10\times 10\;{\text{cm}}^{2}$. Solid line represents diode measurement and dashed line represents Monte Carlo simulated result.

**Figure 4:**
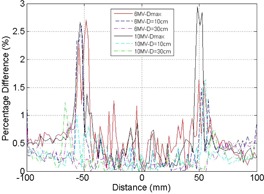
Percentage difference (delta) of OAR between diode measurement and Monte Carlo simulation. Solid line represents delta at depth of maximum dose, dash line represents delta at depth of 10 cm, and dash‐dot line represents delta at depth of 30 cm for 6 MV and 10 MV at 100 SSD with $10\times 10\;{\text{cm}}^{2}$.

### B. SRS cones output factors

**Figure 5:**
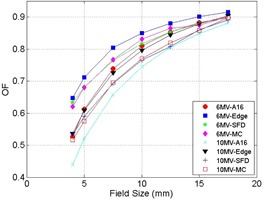
Output factors of SRS cones for 6 MV and 10 MV. The circle represents A16 measurement for 6 MV, the square represents Edge measurement for 6 MV, the star represents SFD measurement for 6 MV, the diamond represents Monte Carlo simulation for 6 MV, the X represents A16 measurement for 10 MV, the triangle represents Edge measurement for 10 MV, the plus represents SFD measurement for 10 MV, and the clear square represents Monte Carlo simulation for 10 MV. Output factors are normalized to a $5\times 5\;{\text{cm}}^{2}$ reference field of the respective energies.

The output factors as a function of SRS cone diameter for A16 ionization chamber, Edge detector, SFD detector, and Monte Carlo are shown on Table 1 for 6 MV and Table 2 for 10 MV. The output factors are normalized to a $5\times 5\;{\text{cm}}^{2}$ reference field. In addition, these output factors are illustrated in Fig. 5 for both energies. In order to demonstrate that the simulated voxel volume is sufficient in order to avoid volume averaging effect, Table 3 compares the output factors generated for voxel volumes of $0.5\times 0.5\times 0.5\;{\text{mm}}^{3}$ and $0.25\times 0.25\times 0.25\;{\text{mm}}^{3}$ for the 5 mm and 4 mm diameter applicators. The differences in these output factors are less than 1% and within simulation relative uncertainty, suggesting that there is no volume averaging effect for $0.5\times 0.5\times 0.5\;{\text{mm}}^{3}$ voxel volume.

**Table 1:** Output factors of SRS cones for 6 MV. Output factors are normalized to a $5\times 5\;{\text{cm}}^{2}$ reference field.

*Cone (mm)*	*A16*	*Edge*	*SFD*	*MC*
17.5	0.898	0.915	0.903	0.901
15.0	0.883	0.901	0.880	0.874
12.5	0.853	0.880	0.854	0.865
10.0	0.810	0.850	0.817	0.831
7.5	0.739	0.804	0.764	0.767
5.0	0.612	0.712	0.682	0.680
4.0	0.526	0.648	0.633	0.617

**Table 2:** Output factors of SRS cones for 10 MV. Output factors are normalized to a $5\times 5\;{\text{cm}}^{2}$ reference field.

*Cone (mm)*	*A16*	*Edge*	*SFD*	*MC*
17.5	0.881	0.904	0.891	0.892
15.0	0.849	0.879	0.861	0.852
12.5	0.805	0.844	0.808	0.814
10.0	0.745	0.796	0.766	0.765
7.5	0.658	0.726	0.693	0.692
5.0	0.521	0.609	0.590	0.571
4.0	0.439	0.536	0.533	0.510

**Table 3:** Monte Carlo output factors voxel volume comparison.

*Energy, Cone (mm)*	*0.5 mm^3^*	*0.25 mm^3^*	*% Difference*
6 MV, 5.0	0.680	0.674	0.88
6 MV, 4.0	0.617	0.611	0.97
10 MV, 5.0	0.571	0.573	0.35
10 MV, 4.0	0.510	0.506	0.78

## IV. DISCUSSION

The output factor values of A16 ionization chamber illustrate a partial volume effect for cone sizes smaller than 12.5 mm chiefly due to its 4.4 mm collector length. The higher output factor values of Edge and SFD detectors suggest an overresponse due to the use of diode detectors. It is known that the unshielded silicon diode detector has an over response to low‐energy secondary scattered photons due to the photoelectric effect.^14^, ^21^ There are a greater quantity of lower energy photons present in the flattening filter‐free energy spectrum when compared with a flattened energy spectrum. This effect is even more pronounced for small radiation fields. A simple method of detector correction under such circumstances is the “daisy‐chaining” method.^(22)^ This uses an intermediate field size measurement for both ionization chamber and diode in order to correct the output factors; however, since the daisy‐chaining method is only limited to corrections relating the reference field to the intermediate field, the detector correction does not take into account of all of the effects of small field dosimetry.

**Figure 6:**
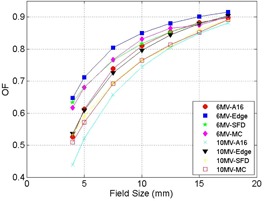
Corrected output factors of SRS cones for 6 MV and 10 MV. The square represents Edge measurement for 6 MV, the star represents SFD measurement for 6 MV, the diamond represents Monte Carlo simulation for 6 MV, the triangle represents Edge measurement for 10 MV, the plus represents SFD measurement for 10 MV, and the clear square represents Monte Carlo simulation for 10 MV. Output factors are normalized to a $5\times 5\;{\text{cm}}^{2}$ reference field of the respective energies.

For comprehensive correction, Alfonso et al.^(23)^ proposed a detector correction factor, $k_{Q_{\text{clin}},Q_{\text{msr}}}^{f_{\text{clin}},f_{\text{msr}}}$, that accounts for the differences between detector responses in the clinical radiation field $f_{\text{clin}}$ for which the absorbed dose to water needs to be determined with beam quality $Q_{\text{clin}}$, and in the machine‐specific reference field $f_{\text{msr}}$ with beam quality $Q_{\text{msr}}$. Francescon et al.^(24)^ reported $k_{Q_{\text{clin}},Q_{\text{msr}}}^{f_{\text{clin}},f_{\text{msr}}}$ corrections of several detectors using Monte Carlo simulations for two 6 MV accelerators: the Primus (manufactured by Siemens) and the Synergy (manufactured by Elekta). In their study, the MC simulated results of both accelerators are compared with measurements using several small detectors to determine the detector correction factors. They reported that Edge detector correction factors for 5 mm, 7.5 mm, 10 mm, 12.5 mm, and 15 mm square fields are 0.933, 0.952, 0.966, 0.976, and 0.983, respectively, for Primus, and 0.922, 0.950, 0.968, 0.980, and 0.988, respectively, for Synergy. Although these correction factors from the Francescon study are informative, their usage is limited in our study due to differences in energy spectrums between unflattened beam and the flattened filtered beam. In addition the differences between accelerator manufacturers, as well as different models produced by the same vendor, can contribute to different energy spectra, even if mechanical components are the same. Tanny et al.^(25)^ reported a $k_{Q_{\text{clin}},Q_{\text{msr}}}^{f_{\text{clin}},f_{\text{msr}}}$ correction factor study using multiple detectors, including the Edge diode detector, for Varian TrueBeam with flattening filter‐free energies of 6 MV (6 MV‐FFF) and 10 MV (10 MV‐FFF). Measurements of square field sizes from $6\times 6\;{\text{mm}}^{2}$ to $5\times 5\;{\text{cm}}^{2}$ with the Edge detector were compared to measurements from Exradin W1 scintillating fiber optic dosimeter manufactured by Standard Imaging. For 6 MV‐FFF, Edge detector has correction factors of 0.949, 0.963, 0.977, 0.988, and 0.997 for 6 mm, 8 mm, 10 mm, 12 mm, and 14 mm square field, respectively. For 10 MV‐FFF, Edge detector has correction factors of 0.901, 0.929, 0.944, 0.957, and 0.966 for 6 mm, 8 mm, 10 mm, 12 mm, and 14 mm square field, respectively. These corrections from the Tanny study are applied to the cone sizes in our study through interpolation and extrapolation. The correction factors for Edge detector with respect to 6 MV‐FFF are 0.935, 0.942, 0.960, 0.977, 0.990, 0.999, and 1.00 for 4 mm, 5 mm, 7.5 mm, 10 mm, 12.5 mm, 15 mm, and 17.5 mm size cones, respectively. For 10 MV‐FFF, the Edge correction factors are 0.873, 0.887, 0.922, 0.944, 0.959, 0.969, and 0.975 for 4 mm, 5 mm, 7.5 mm, 10 mm, 12.5 mm, 15 mm, and 17.5 mm size cone, respectively. The output factors after the application of these correction factors to the Edge detector, as well as percentage differences with MC simulation results, are illustrated in Fig. 6, as well as 4, 5 for 6 MV and 10 MV, respectively. After correction, the Edge detector output factors are within 3% of MC results for 6 MV. However, for 10 MV, the Edge output factors are significantly lower than MC results for cone diameters of 7.5 mm and less. One possible source of error which may explain this is that the correction factors for 4 mm and 5 mm were extrapolated from data by Tanny et al.^(25)^ and not actually measured. Another possible source of error is the different measurement conditions utilized by our study, relative to the one by Tanny and colleagues. In that study, measurements were performed at 100 cm SSD and at a depth of 10 cm; while in our study, measurements were performed at an SSD 95 cm and 5 cm depth. In addition, the Tanny study was conducted utilizing square field sizes, while the present study was conducted using circular field sizes.

**Table 4:** Corrected output factors of SRS cones for 6 MV. Corrections for over responses of Edge and SFD diode detectors have been applied. The percentage differences between Edge and SFD with MC are illustrated in the final two columns.

*Cone (mm)*	*Edge*	*SFD*	*MC*	*% Diff Edge/MC*	*% Diff SFD/MC*
17.5	0.915	0.903	0.901	1.55	0.22
15.0	0.900	0.880	0.874	2.97	0.69
12.5	0.871	0.854	0.865	0.69	1.27
10.0	0.830	0.811	0.831	0.12	2.41
7.5	0.771	0.750	0.767	0.52	2.22
5.0	0.671	0.658	0.680	1.32	3.24
4.0	0.606	0.595	0.617	1.78	3.57

**Table 5:** Corrected output factors of SRS cones for 10 MV. Corrections for over responses of Edge and SFD diode detectors have been applied. The percentage differences between Edge and SFD with MC are illustrated in the final two columns.

*Cone (mm)*	*Edge*	*SFD*	*MC*	*% Diff Edge/MC*	*% Diff SFD/MC*
17.5	0.881	0.891	0.892	1.23	0.11
15.0	0.852	0.861	0.852	0.0	1.06
12.5	0.810	0.808	0.814	0.49	0.74
10.0	0.751	0.761	0.765	1.83	0.52
7.5	0.669	0.681	0.692	3.32	1.59
5.0	0.540	0.570	0.571	5.43	0.18
4.0	0.468	0.501	0.510	8.24	1.76

Although there is currently no reported $k_{Q_{\text{clin}},Q_{\text{msr}}}^{f_{\text{clin}},f_{\text{msr}}}$ correction factor of SFD detector for TrueBeam 6 MV‐FFF or 10 MV‐FFF, there are studies conducted with flatten 6 MV accelerators. Ralston et al.^(26)^ reported small field diode correction factors using fiber optic scintillation dosimeter with EBT2 radiochromic film as a reference for 6 MV stereotactic cones of diameter 4–30 mm from a Varian Novalis‐Tx accelerator. In that study, correction factors for detectors, including SFD and PFD, are presented at depths of 1.5 cm, 5 cm, and 10 cm for two different detector orientations (stem is either parallel or perpendicular to the axis of the beam). The reported correction factors for the SFD detector at a depth of 5 cm in parallel orientation (which is the condition closest to our study) are 0.93, 0.97, 0.98, and 0.99 for 4 mm cone, 5 mm MLC, 7.5 mm cone, and 10 mm cone, respectively. Correction factors for the SFD appear to be near 1.00 for cone sizes greater than 10 mm. It should be noted that the 6 MV spectrum in the Varian Novalis‐Tx is harder due to the presence of a flattening filter. The overresponse in the diode measurements of our study is expected to be greater, due to the increased presence of lower energy photons because of the lack of flattening filter. Cranmer‐Sargison et al.^(11)^ also reported correction factors of SFD detector by using Monte Carlo simulation. MC simulations (EGSnrc) were benchmarked for the Varian Clinac iX 6 MV accelerator to determine $k_{Q_{\text{clin}},Q_{\text{msr}}}^{f_{\text{clin}},f_{\text{msr}}}$ correction factors for various diode detectors. Correction factors for detectors were presented for square field sizes between 0.45 cm to 5.0 cm at depths of 1.5 cm, 5 cm, and 10 cm. Their correction factors for the SFD detector at depth of 5 cm are 0.955, 0.961, 0.984, and 0.996 for 4.5 mm, 5 mm, 7.5 mm, and 10 mm square fields, respectively. The correction factors for SFD detector also appear to be 1.00 for field sizes greater than 10 mm. Although there was no machine‐specific correction factor for the SFD detector in this study, significant error may result if detector correction factors are not applied in small field dosimetry. This study averaged the SFD detector correction factors by Ralston et al.^(26)^ and Cranmer‐Sargison et al.^(11)^ resulting in 0.9395, 0.9655, 0.982, and 0.993 for 4 mm, 5 mm, 7.5 mm, and 10 mm cone, respectively. No correction factors were applied for cone size greater than and equal to 12.5 mm. The output factors after the application of these correction factors to SFD detector, as well as percentage differences with MC simulation results, are illustrated in Fig. 6 as well as 4, 5 for 6 MV and 10 MV, respectively. When no correction factors are needed such as for cone sizes greater than and equal to 12.5 mm, the output factors between SFD and MC are within 1.5% for both energies. For cone sizes less than 12.5 mm, the differences of output factors between SFD and MC for 6 MV after detector correction increase to as much as 3.5% for 4 mm cone. The output factors of SFD are actually lower than the MC values after detector correction. The correction factors were determined for a harder energy spectrum due to presence of flattening filter. Clearly the differences in energy spectrum are insufficient to account for the differences in output factors between SFD and MC, since a softer spectrum would require greater detector correction. One possible reason for this may be the difference in measurement conditions. In both the Ralston and Cranmer‐Sargison studies, measurements were performed at 100 cm SSD while, in our study, measurements were performed at 95 cm SSD. In addition, different accelerator designs and components are likely to yield different correction factors. For 10 MV, with cone sizes less than 12.5 mm, the difference of output factors between SFD and MC after detector correction is less than 2%. This agreement is fortuitous since the correction factors were determined with different energy, machine, and measurement conditions. Future investigation on machine‐specific correction factors is warranted to elucidate the differences of output factors.

## V. CONCLUSIONS

This study presents the output factors of Varian SRS system for the seven conical collimators of 17.5 mm, 15.0 mm, 12.5 mm, 10.0 mm, 7.5 mm, 5.0 mm, and 4.0 mm by using diodes measurements and Monte Carlo simulation for 6 MV and 10 MV. Significant error may result if detector correction factors are not applied in small field dosimetry. Although it lacked the machine‐specific $k_{Q_{\text{clin}},Q_{\text{msr}}}^{f_{\text{clin}},f_{\text{msr}}}$ correction factor for diode detectors, correction factors were applied utilizing published studies conducted under similar conditions. For cone diameters greater than or equal to 12.5 mm, the output factors between Edge detector, SFD detector, and MC simulations are within 3.0% for both energies. For cone diameters less than 12.5 mm, output factors between Edge, SFD, and MC have greater variations.

## ACKNOWLEDGMENTS

The authors would like to thank Varian Medical Systems for providing detailed information of the Stereotactic Radiosurgery System and TrueBeam linear accelerator. This research was supported by the Intramural Research Program of the National Cancer Institute, NIH.

## COPYRIGHT

This work is licensed under a Creative Commons Attribution 4.0 International License.

## Supporting information

Supplementary Material

Supplementary Material

Supplementary Material

Supplementary Material

Supplementary Material

Supplementary Material

Supplementary Material

Supplementary Material

Supplementary Material

Supplementary Material
