# Supplementary figures and images for: Output factor comparison of Monte Carlo and measurement for Varian TrueBeam 6 MV and 10 MV flattening filter‐free stereotactic radiosurgery system
Source: J Appl Clin Med Phys. 2016 May 8;17(3):100–10. doi: 10.1120/jacmp.v17i3.5956 (PMC5690931; doi:10.1120/jacmp.v17i3.5956)

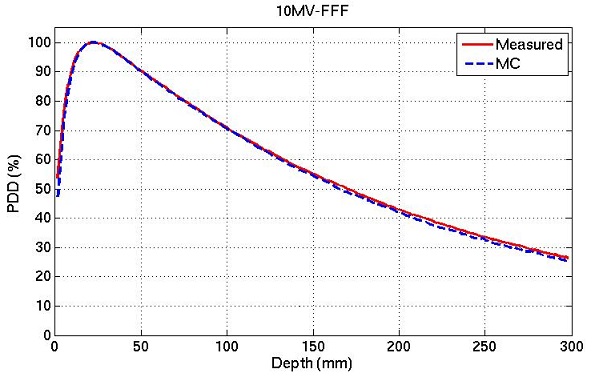

Supplement: Supplementary file 1 — Supplementary Material [file ACM2-17-100-s001.jpg]

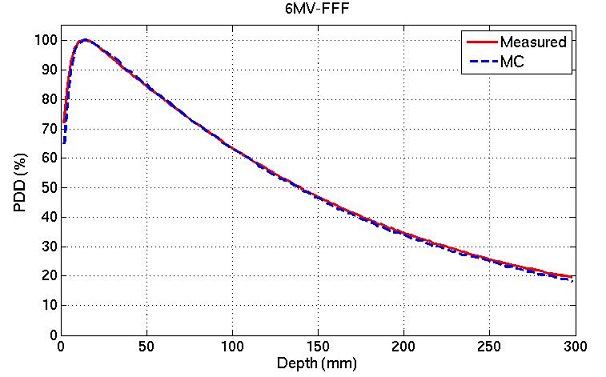

Supplement: Supplementary file 2 — Supplementary Material [file ACM2-17-100-s002.jpg]

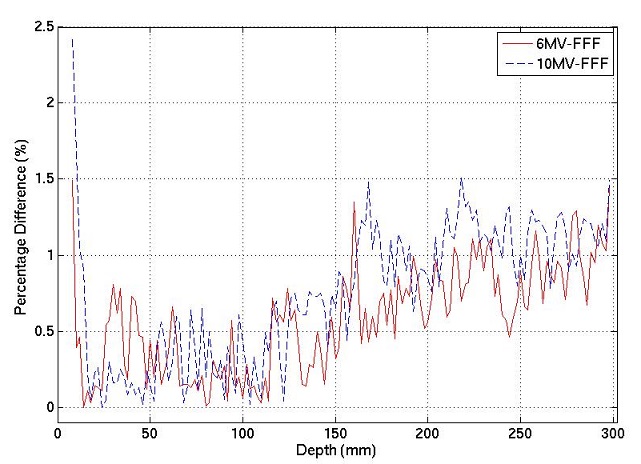

Supplement: Supplementary file 3 — Supplementary Material [file ACM2-17-100-s003.jpg]

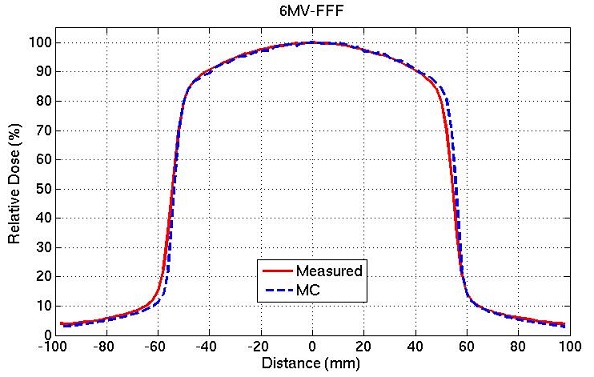

Supplement: Supplementary file 4 — Supplementary Material [file ACM2-17-100-s004.jpg]

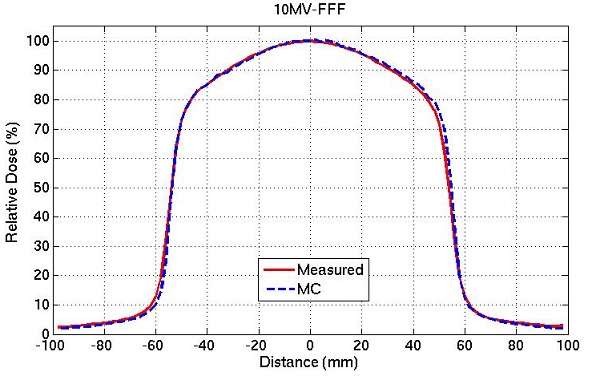

Supplement: Supplementary file 5 — Supplementary Material [file ACM2-17-100-s005.jpg]

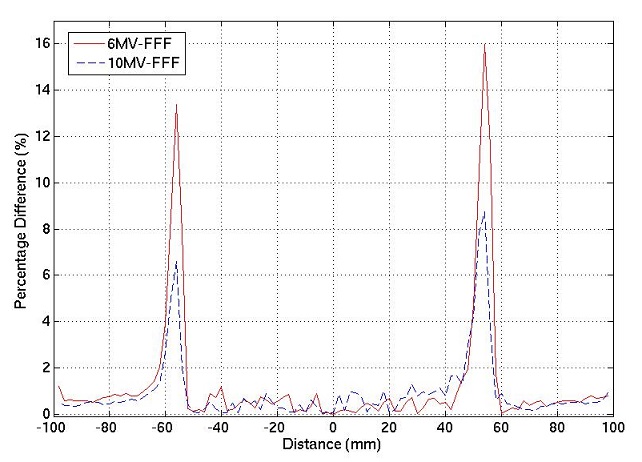

Supplement: Supplementary file 6 — Supplementary Material [file ACM2-17-100-s006.jpg]

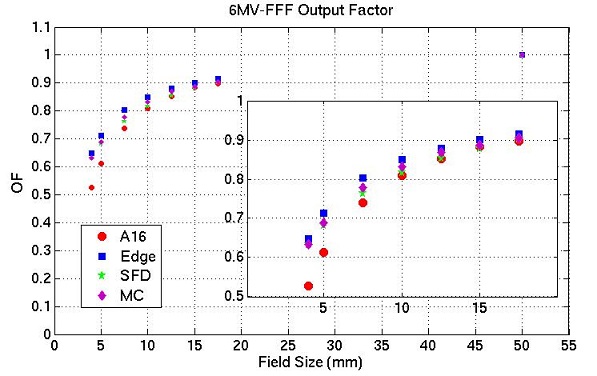

Supplement: Supplementary file 7 — Supplementary Material [file ACM2-17-100-s007.jpg]

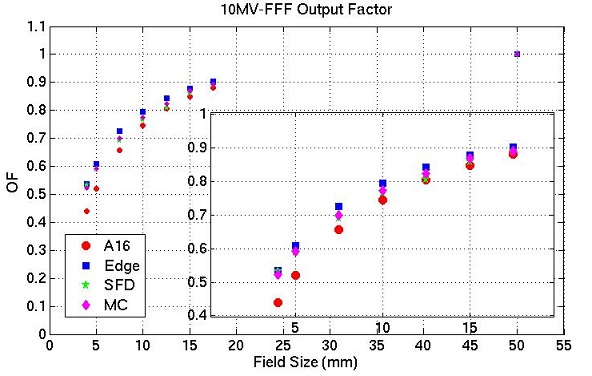

Supplement: Supplementary file 8 — Supplementary Material [file ACM2-17-100-s008.jpg]

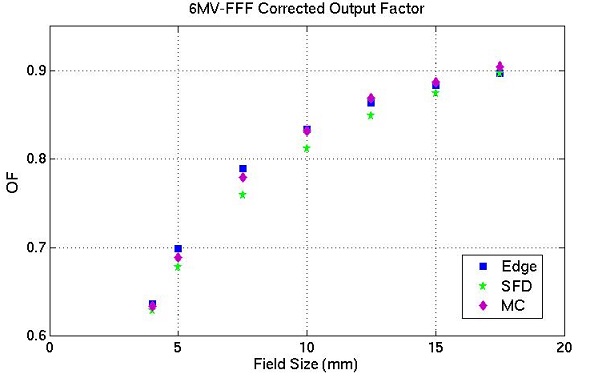

Supplement: Supplementary file 9 — Supplementary Material [file ACM2-17-100-s009.jpg]

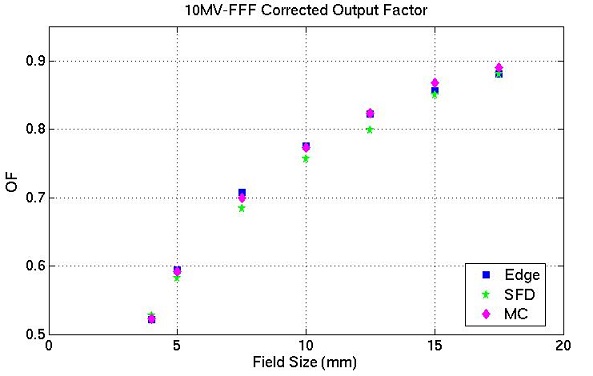

Supplement: Supplementary file 10 — Supplementary Material [file ACM2-17-100-s010.jpg]
